# Identification of immune cells infiltrating in hippocampus and key genes associated with Alzheimer’s disease

**DOI:** 10.1186/s12920-023-01458-2

**Published:** 2023-03-13

**Authors:** Chenming Liu, Sutong Xu, Qiulu Liu, Huazhen Chai, Yuping Luo, Siguang Li

**Affiliations:** 1 Key Laboratory of Spine and Spinal Cord Injury Repair and Regeneration of Ministry of Education, Orthopedic Department of Tongji Hospital, Tongji University School of Medicine, Shanghai, 200092 China; 2 Stem Cell Translational Research Center, Tongji Hospital, Tongji University School of Medicine, Shanghai, 200065 China

**Keywords:** Alzheimer’s disease, hippocampus, Immune infiltration, Key genes

## Abstract

Alzheimer’s disease (AD) is the most prevalent cause of dementia and is primarily associated with memory impairment and cognitive decline, but the etiology of AD has not been elucidated. In recent years, evidence has shown that immune cells play critical roles in AD pathology. In the current study, we collected the transcriptomic data of the hippocampus from gene expression omnibus database, and investigated the effect of immune cell infiltration in the hippocampus on AD, and analyzed the key genes that influence the pathogenesis of AD patients. The results revealed that the relative abundance of immune cells in the hippocampus of AD patients was altered. Of all given 28 kinds of immune cells, monocytes were the important immune cell associated with AD. We identified 4 key genes associated with both AD and monocytes, including KDELR1, SPTAN1, CDC16 and RBBP6, and they differentially expressed in 5XFAD mice and WT mice. The logistic regression and random forest models based on the 4 key genes could effectively distinguish AD from healthy samples. Our research provided a new perspective on immunotherapy for AD patients.

**Supplementary Information:**

The online version contains supplementary material available at 10.1186/s12920-023-01458-2.

## Background

Alzheimer’s disease (AD) is an age-related neurodegenerative disorder that primarily involves memory decline and executive dysfunction. The main features of AD are abnormal aggregation of extracellular amyloid plaques and hyperphosphorylation of neuronal tau, which lead to synaptic loss and neuronal atrophy [1]. Experts now believe that, like other common chronic diseases, AD is caused by a combinational factor [2], including age, environment, genetics, or specific susceptibility genes [3–5]. Cardiovascular disease, diabetes, obesity, and diet are generally considered to be factors that increase the risk of AD [6–8]. Activated microglia and astrocytes in AD patient brains usually have higher levels of inflammatory markers, which are generally distributed around amyloid plaques and neurofibrillary tangles [9, 10]. The role of immune response in the brain of AD patients may be bidirectional. On the one hand, pathogenic substances such as cell debris and protein aggregates can be eliminated by phagocytosis of microglia and astrocytes; On the other hand, persistent neuroinflammation is a chronic response of the innate immune system to neurological changes, and the sustained activation of glial cells causes harm to the nervous system [11]. In cell culture studies, activated microglia could produce harmful substances, which may damage neurons [12–14]. Another feature of AD is the impairment of the blood brain barrier (BBB), and a compromised BBB might increase the permeability of immune cells and peripheral tissue molecules, which could lead to neurodegeneration [15]. Both peripheral macrophages and neutrophils can infiltrate the brain of AD patients through the BBB and induce the activated innate immune response in AD patients [16–18]. In addition, activated T cells are also found in the brain of AD patients, where they could release inflammatory factors[19–21]. Amyloid β (Aβ), which aggregates alone, has been found to be a powerful complement activator [22]. Activation of the complement system in AD patients results in the production of allergenic toxins that further promote inflammation [23], cytokine-induced APP production, and higher Aβ production due to increased APP amounts [24, 25]. Although numerous studies have shown that inflammation plays a vital role in the pathogenesis of AD, the identification of immune cells closely related to AD and the molecular mechanisms of AD pathogenesis requires further elucidation.

In this study, we assess the level of immune infiltration from the hippocampus based on the expression of given immune cell genes by single sample gene set enrichment analysis (ssGSEA), and revealed the differences in the immune infiltration of hippocampal tissue in AD and healthy samples. We identified key genes from highly correlated co-expression modules, which were closely associated with disease and immune cells. This study laid the foundation for further finding effective targets for curing AD and developing immunomodulatory regimens for effective treatment of AD.

## Materials and methods

### Data preprocessing and immune infiltration assessment

At first, we used “hippocampus” and “Alzheimer’s disease” as keywords to search the datasets in the GEO database, and we found the GSE5281 and GSE48350 datasets, which were both from the GPL570 platform. The ssGSEA could assess the infiltration of 28 immune cells for each AD and control sample through GSEA package [26]. We retained the immune cells with significant differences as traits for subsequent analysis (*p* value < 0.05).

## Weighted gene co-expression network analysis (WGCNA)

At first, we used the limma package to normalize the raw data of all samples, and then we removed genes containing NA. We used WGCNA package to construct a gene co-expression network to find key modules and module genes [27]. Genes were clustered based on the phase dissimilarity machine. The division of modules was based on the high topological overlap of genes within the modules [28]. We selected the modules associated with disease for subsequence analysis. For genes within modules, we further screened based on gene significance (GS) and module importance (MM). The genes with high MM and high GS were described as the central module genes, which were strongly associated with disease and candidate immune cell. In our study, the central module genes were the genes in the candidate module with |MM| > 0.8 and |GS| > 0.2.

## Functional enrichment analysis

Gene Ontology (GO) and Kyoto Encyclopedia of Genes and Genomes (KEGG) pathway analysis of central module genes were conducted by clusterProfiler [29] and ReactomePA packages [30].

## Analysis of protein–protein interaction (PPI) network and identification of key genes

The Search Tool for the Retrieval of Interacting Genes (STRING) online tool [31] was used to analyze the PPI of central module genes with the default parameters. Then we used the cytoHubba plugin [32] of the Cytoscape (version 3.8.2) to identify the key genes [33]. The cytoHubba provides 12 analysis algorithms to calculate hub genes in protein interaction network graphs, we used five of which to identify key genes in the PPI network, including Degree, Edge Penetration Component (EPC), Maximum Neighborhood Component (MNC), Density of Maximum Neighborhood Component (DMNC) and Maximum Group Centrality (MCC). We regarded the intersection of top10 genes, which were obtained by cytoHubba’s five algorithms as the key genes, the VennDiagram package was used to visualize these results [34].

## Validation of the key genes

We constructed logistic regression model and random forest model by the intersection genes of cytoHubba’s five algorithms to explore the correlation between disease and key genes. We randomly divided all samples into test and training cohort according to the proportion of 3/7, we generated logistic regression model and random forest model in the training cohort and validated the performance of the models in the test cohort. The receiver operating characteristic (ROC) curves and confusion matrix were used to assess the validity of the models [35].

## Animals

5.5-month-old heterozygous 5XFAD mice are housed in Tongji University Animal Center under standard conditions. Aβ42 began to accumulate in the brains of 5XFAD mice at 1.5 months of age [36]. There are many Aβ plaques in the hippocampus at 5.5 months of age. In this study, 5.5-month-old 5XFAD mice were euthanized and hippocampal tissue was isolated for subsequent experiments. Both AD and control groups contained two female mice and two male mice.

## RNA extraction and quantitative real-time PCR (qRT-PCR)

The total RNA of the hippocampus in all mice were extract by RNAiso Plus (9109, TaKaRa, China). According to the manufacturer’s instructions, qRT-PCR was performed by the AceQ Universal SYBR qPCR Master Mix (Q511-02, Vazyme, China). All genes’ expression levels were normalized to β-Actin by the comparative CT method (2^−ΔΔCt^). Table 1 showed the sequences of all RNA primers.

**Table 1:** The primer sequences used for RT-qPCR.

Genes	Sequences
β-actin	Forward: CTAAGGCCAACCGTGAAAAG Reverse: ACCAGAGGCATACAGGGACA
Kdelr1	Forward: GTGGTGTTCACTGCCCGATA Reverse: AACTCCACCCGGAAAGTGTC
Sptan1	Forward: ACAAGGACCCCACCAACATC Reverse: GCCTTGACAGCATCCTCACT
Cdc16	Forward: CCTGTGTCTTGGTTTGCGGT Reverse: TCTCCACAGCGAAGGAATGC
Rbbp6	Forward: TTAGCATGAGCGAGTGGGAC Reverse: ACAACGAAGGACCCTAAGGC

### Statistical analysis

All data were visualized and analyzed by GraphPad Prism 8. *T* test was used to compare expression level between the AD and WT groups and *p* value < 0.05 were considered statistically significant.

## Gene set enrichment analysis (GSEA)

Based on the median expression levels of key genes, we divided all samples into high and low expression groups, and GSEA was performed to explore hallmark pathways between the two groups [37]. We used *p* value < 0.05 and p-adjust < 0.25 as the screening criterion for statistically significant.

## Construction of mRNA-miRNA-lncRNA network

To further explore the miRNA and lncRNA regulatory networks associated with key genes, we constructed an mRNA-miRNA-lncRNA network based on the key genes screened in the previous result. At first, based on the “multiMiR” package [38], we used experimentally validated data to explore miRNAs associated with key genes. After obtaining miRNAs that interact with key genes, we used the starBase database to explore lncRNAs that interact with miRNAs [39]. The lncRNAs that interact weakly with miRNAs are removed. Our screening criteria were that miRNA-lncRNA expression was negatively correlated in more than four cancers and validated by more than three clip-seq experiments. Finally, we used Cytoscape for visualization of the mRNA-miRNA-lncRNA network.

## Result

### Data processing

**Fig. 1:**
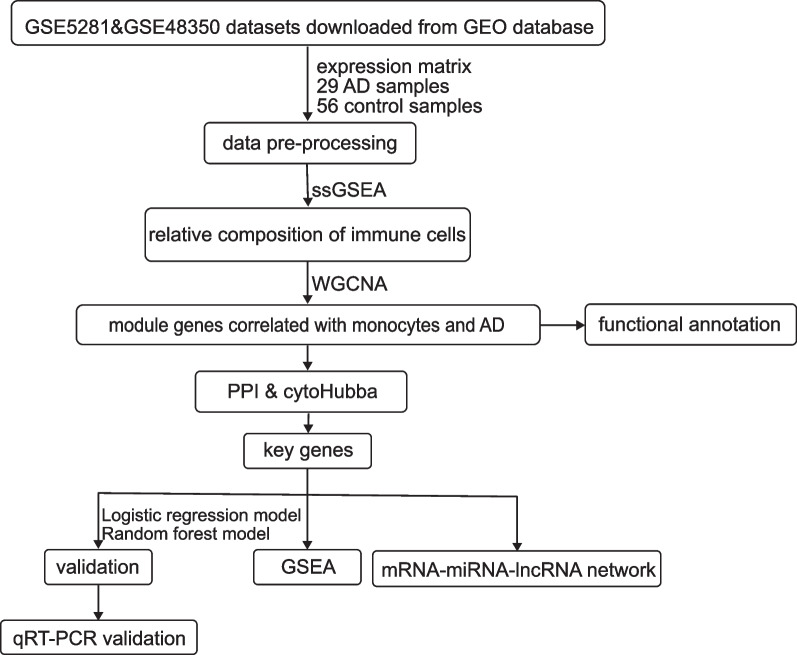
Flow chart of this study

This study procedure was conducted methodically based on the steps outlined in the flow diagram (Fig. 1). Based on the search for keywords in Materials and Methods, we downloaded two datasets, GSE5281 and GSE48350, from the GEO database. As we mainly focused on the changes in transcriptome data of hippocampal tissue, we selected the data of hippocampal tissue, GSE5281 containing 23 samples and GSE48350 containing 62 samples. The R software was used to process the raw expression profiles of these two datasets, and the limma package was used to normalize the raw data [40]. The batch effects of these two datasets were processed by the “sva” package [41].

## Immune infiltration in the hippocampus of AD patients might be altered

**Fig. 2:**
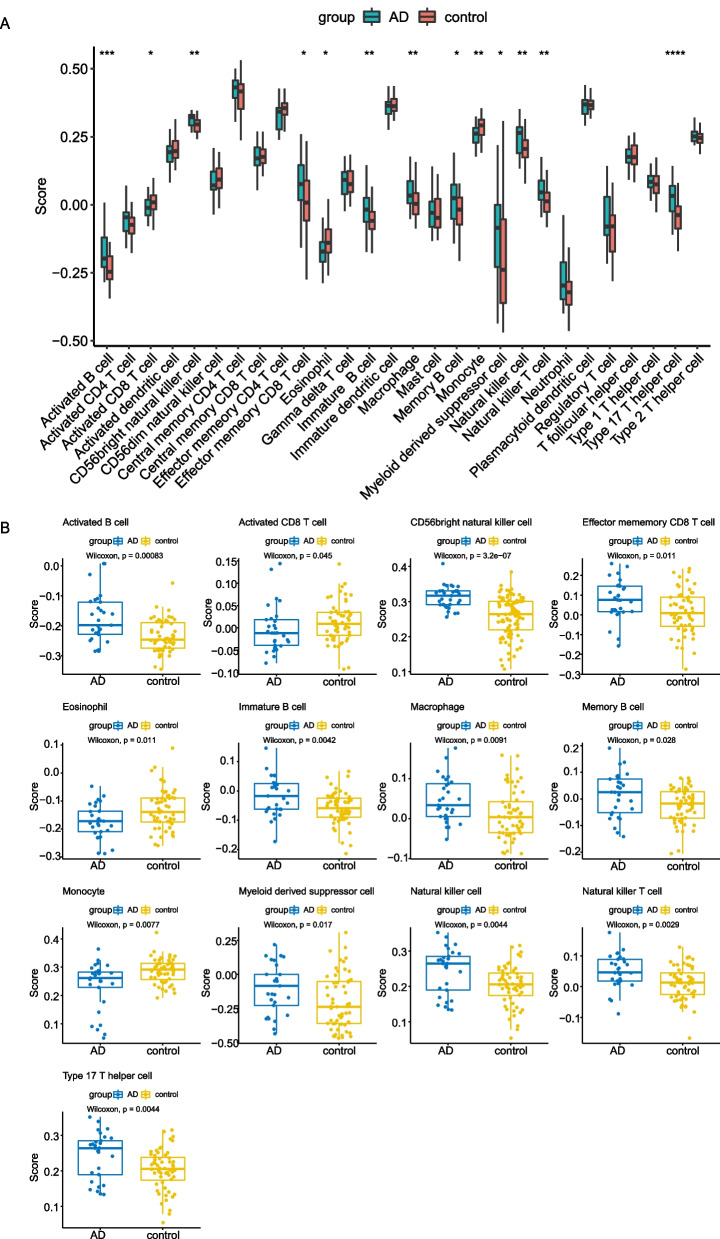
Immune infiltration analysis. **A** Boxplot of the enrichment score of 28 immune cells in each AD and healthy sample. **B** The immune cells with significant differences between AD and healthy samples

As described in methods and materials, ssGSEA was performed on 29 AD samples and 56 control samples to assess the scores of 28 immune cells (Fig. 2A, B). Our results indicated that the scores of activated B cell, activated CD8 T cell, CD56 bright natural killer cell, effector memory CD8 T cell, eosinophil, immature B cell, macrophage, memory B cell, monocyte, myeloid derived suppressor cell, natural killer cell, natural killer T cell and type 17 T helper cell were significantly different between AD and healthy groups (*p* < 0.05), indicating that the level of immune cell infiltration might be altered in the hippocampus of AD patients.

## Monocytes were the important immune cell associated with AD in the hippocampus

**Fig. 3:**
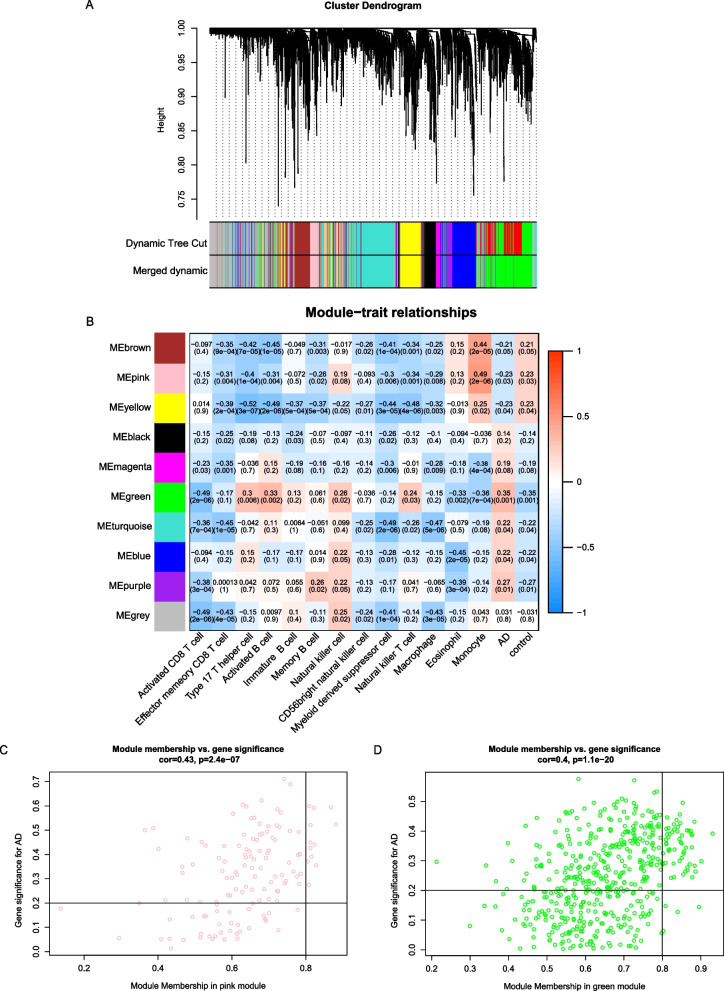
Identification of key modules correlated with AD and monocytes in the datasets through WGCNA. **A** Cluster dendrogram of all genes. **B** The heatmap showed relationship of each module and traits. **C** Scatterplot of genes in the pink module. **D** Scatterplot of genes in the green module

To identify disease-associated immune cell types associated with disease, we constructed gene co-expression modules using WGCNA. We first normalized the data from the datasets and subsequently removed genes containing NA. 2971 genes were eligible for further analysis. We built a scale-free (scale-free R2 > 0.85) co-expression network using soft threshold power β = 12 (Additional file 1: Fig. S1). These 2971 genes were clustered into 10 different color modules (Fig. 3A, B). Then, we analyzed the correlation between each module and immune cell types or sample types (AD and control) (Figure B). As a result, the green module was positively correlated with AD but negatively correlated with monocytes, and in contrast, the pink module was negatively correlated with AD but positively correlated with monocytes (*p* < 0.05). Additionally, monocytes exhibited highly correlation with both pink and green module. These results suggested that monocytes infiltrating the hippocampus might be the important immune cell associated with AD.

### Pink and green central module genes were mainly involved in lipid metabolism, notch signaling pathway and material transport by Golgi-associated vesicles transport.

**Fig. 4:**
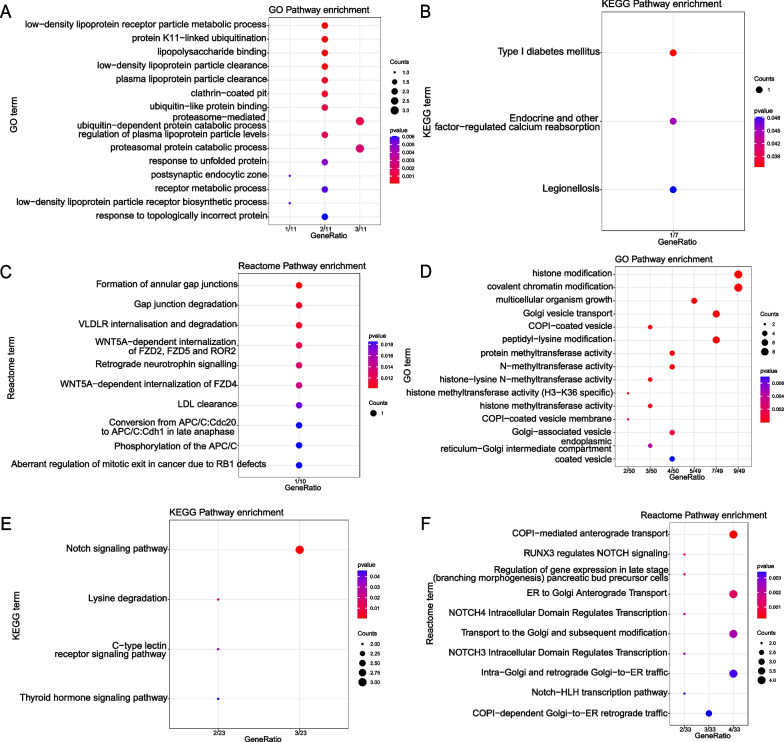
Functional enrichment analysis of pink and green central module genes. **A** The GO result of central pink module genes. **B** The KEGG result of central pink module genes [42]. **C** The Reactome result of central pink module genes. **D** The GO result of central green module genes. **D** The KEGG result of central green module genes [42]. **F** The Reactome result of central green module genes

13 and 55 genes with high connectivity (|MM| > 0.8 and |GS| > 0.2) were screened from pink and green modules, respectively (Fig. 3C, D), which were considered as central module genes. To clarify the biological processes of the pink and green central module genes, we conducted GO, KEGG and Reactome enrichment analysis. According to our selection criteria, as for GO, the pink central module genes were mainly involved in misfolded protein reactions, proteasome-mediated proteolytic metabolic processes and low-density lipoprotein particle metabolism (Fig. 4A). The KEGG analysis suggested the pink central module genes were mainly involved in type 1 diabetes mellitus, legionellosis and endocrine and other factor-regulated calcium reabsorption (Fig. 4B). Reactome analysis demonstrated that pink central module genes were mainly involved in wnt signaling pathway and lipid metabolism (Fig. 4C). The same analysis was also performed on the green module genes. The GO analysis suggested that the 55 green central module genes were mainly enriched in histone modification and Golgi-associated vesicles transport (Fig. 4D). KEGG analysis revealed that the 55 green central module genes were mainly involved in thyroid hormone signaling pathway, notch signaling pathway, lysine degradation and C-type lectin receptor signaling pathway (Fig. 4E). As for Reactome analysis, green central module genes were mainly focused on notch signaling and the transport of substances between the Golgi and the endoplasmic reticulum (Fig. 4F). As a result, based on the frequency of terms, the pink central module genes were mainly affected lipid metabolism, and the green central module genes were mainly affected notch signaling pathway and material transport by Golgi-associated vesicles transport.

## KEDLR1, SPTAN1, CDC16 and RBBP6 were identified as key genes associated with AD and monocytes

**Fig. 5:**
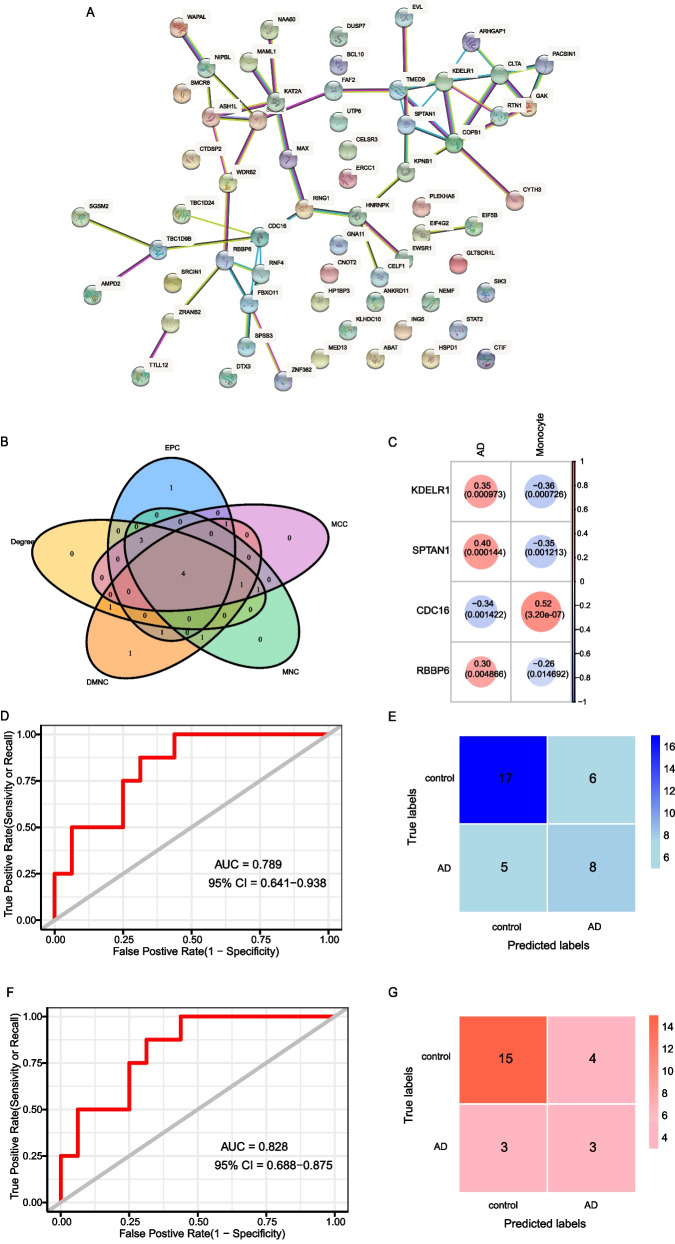
Multiple algorithms identified 4 key genes associated with AD and monocytes. **A** The PPI network of central genes in the pink and green modules. **B** A Venn diagram between five algorithms of cytoHubba. The coincident part represents the four genes (KDELR1, SPTAN1, CDC16 and RBBP6) identified by all five algorithms. The lines between nodes in the PPI network diagram represent the interactions between the nodes. **C** The correlations between 4 key genes and monocytes and AD. **D** ROC curve of logistic regression model could distinguish AD and control samples. **E** Confusion matrix of the logistic regression model in test cohort. **F** ROC curve of the RF model could distinguish AD and control samples. **G** Confusion matrix of the RF model in test cohort

As for all the 68 central genes in the pink and green modules, we explored the PPI of these genes by STRING database, and the result was shown in Fig. 5A. The five algorithms of the cytoHubba, including EPC, MCC, MNC, DMNC and Degree, were used to process the PPI network to identify the top 10 genes (Table 2). KEDLR1, SPTAN1, CDC16 and RBBP6 were regarded as the key genes associated with monocytes and AD, which were the common genes identified by the five algorithms, respectively (Fig. 5B). Correlation analysis showed that KDELR1, SPTAN1 and RBBP6 were positively associated with AD and negatively associated with monocytes, while CDC16 was negatively associated with AD and positively associated with monocytes (Fig. 5C).

To validate correlation between KEDLR, SPTAN1, CDC16 and RBBP6 and AD occurrence, we constructed logistic regression model and random forest model. The area under curve (AUC) of logistic regression model was 0.789 (95% CI = 0.641–0.938), and the AUC of RF model was 0.828 (95% CI = 0.688–0.878) (Fig. 5D, F).

**Fig. 6:**
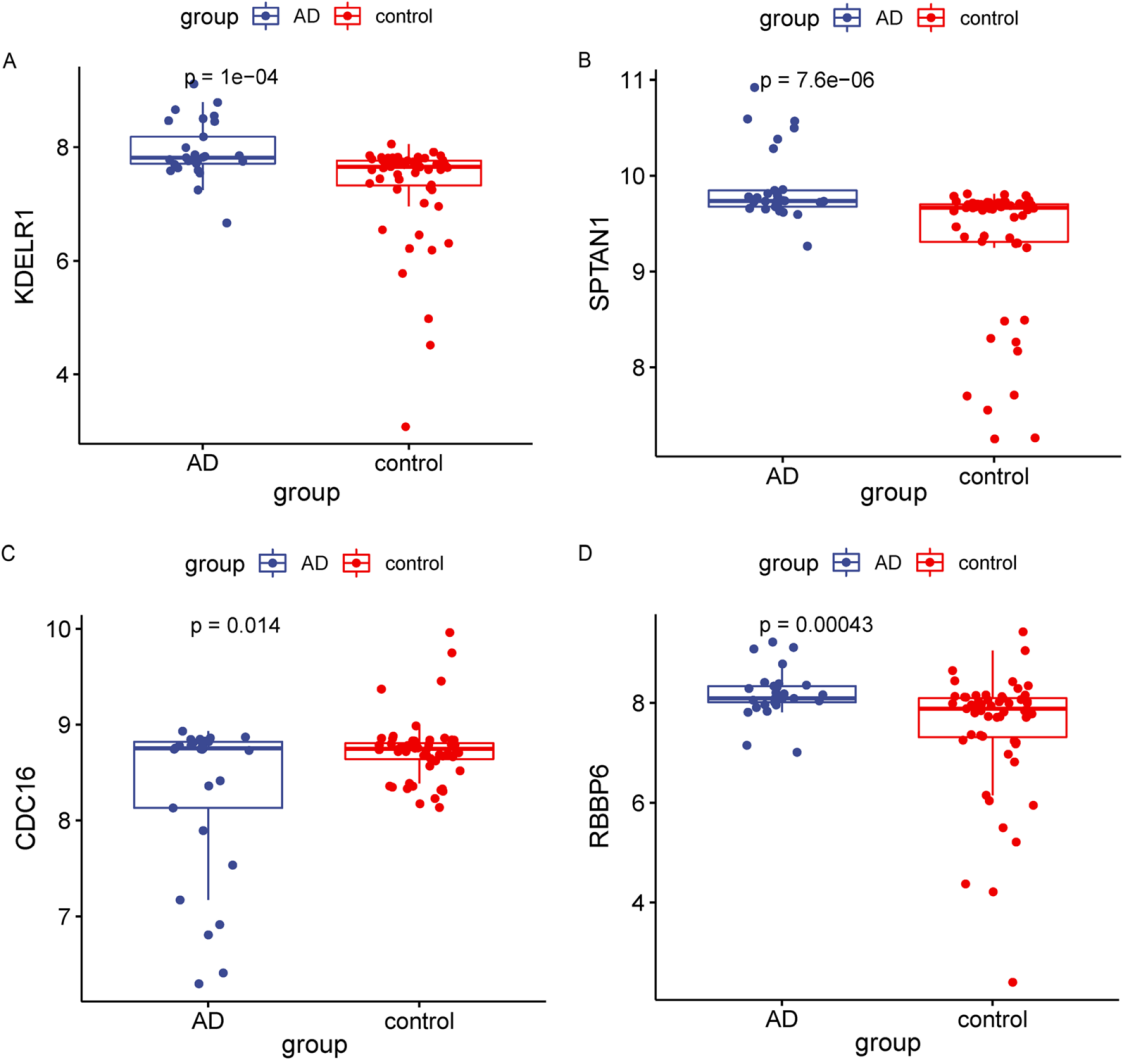
The expression value of the four genes in AD and control samples. **A**–**D** The expression value of KDELR1, SPTAN1, CDC16 and RBBP6 in AD (n = 29) and control (n = 56) samples. Statistical analysis was performed by *t* test

The results of confusion matrix were shown in Fig. 5E, G, and the accuracy and recall of the models were shown in Table 3. These results suggested the logistic regression model and random forest model based on KDELR1, SPTAN1, CDC16 and RBBP6 can distinguish AD patients from healthy samples. Then, we verified the expression values of these 4 genes between the two groups and found that they were significantly different in AD and healthy group (Fig. 6). In summary, multiple algorithms verified KDELR1, SPTAN1, CDC16 and RBBP6 were the key genes corelated with AD.

**Fig. 7:**
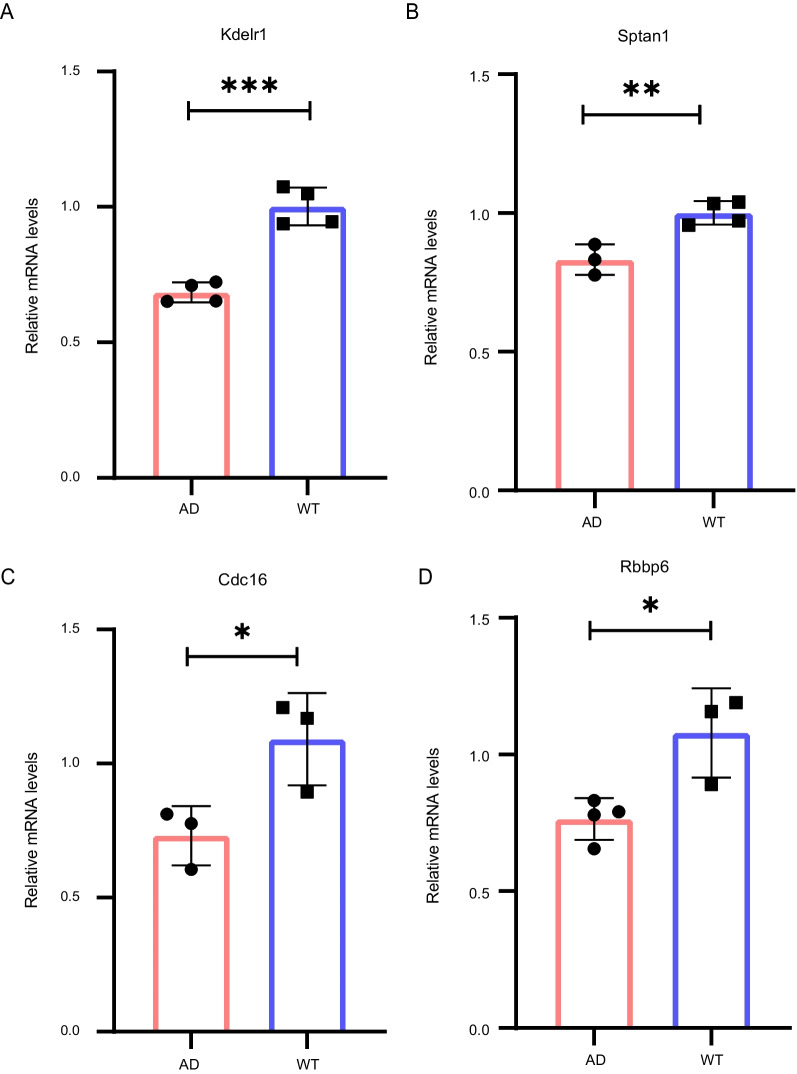
The validation of 4 key genes in 5XFAD and WT mice. **A**–**D** The relative mRNA levels of Kdelr1, Sptan1, Cdc16 and Rbbp6 in WT and 5XFAD mice. ns *p* > 0.05, *p < 0.05, **p < 0.01 and ***p < 0.001. Statistical analysis was performed by *t* test

We also validated the relative mRNA levels of Kdelr1, Sptan1, Cdc16 and Rbbp6 in 5XFAD mice and WT mice. Compared with 5XFAD mice, the relatively mRNA levels of Kdelr1, Sptan1, Cdc16 and Rbbp6 were significantly increased in WT mice (Fig. 7).

**Table 2:** The top 10 genes identified by five algorithms of the cytoHubba, including EPC, MCC, MNC, DMNC and Degree

	Degree	MNC	DMNC	MCC	EPC
1	COPB1	COPB1	CDC16	COPB1	COPB1
2	KDELR1	CLTA	RBBP6	KDELR1	KDELR1
3	CDC16	KDELR1	RNF4	CDC16	SPTAN1
4	KAT2A	ASH1L	FBX011	SPTAN1	TMED9
5	SETD2	SETD2	TMED9	CLTA	CLTA
6	RBBP6	SPTAN1	KDELR1	RBBP6	SETD2
7	CLTA	CDC16	GAK	FBX011	CDC16
8	FBX011	RBBP6	SPTAN1	SETD2	RBBP6
9	SPTAN1	RNF4	ARHGAP1	TMED9	KPNB1
10	ASH1L	FBX011	KAT2A	ASH1L	ASH1L

**Table 3:** The confusion matrix index of logistic regression and random forest models

Index	Logistic regression model	Forest model
	Test cohort	Test cohort
Precision	0.7727	0.8333
Recall	0.7191	0.7895

## GSEA revealed that lipid metabolism and immune response play important roles in AD

**Fig. 8:**
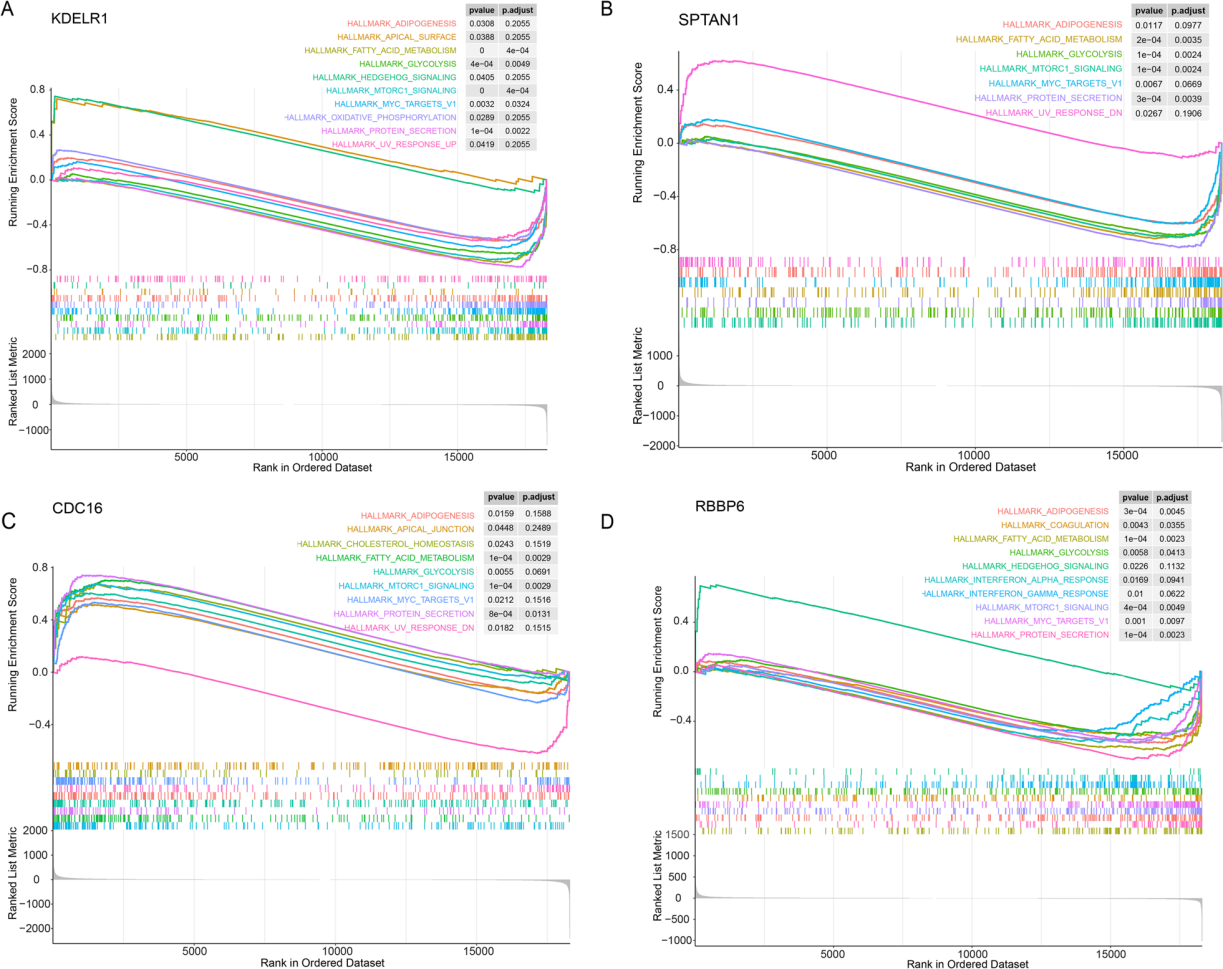
The GSEA results of 4 key genes. **A**–**D** The GSEA results of the group with high expression of KDELR1, SPTAN1, CDC16 and RBBP6

On the basis of the expression value of these 4 key genes, we performed GSEA to explore the potential pathways. We found that samples with high expression of KDELR1, SPTAN1, CDC16 and RBBP6 were enriched for adipogenesis, fatty acid metabolism, glycolysis, mTORc1 signaling, MYC targets V1 and protein secretion proteolysis (Fig. 8). In addition, samples with high expression of KDELR1 were enriched in four other gene sets including apical surface, hedgehog signaling, oxidative phosphorylation and UV response up (Fig. 8A), while samples with high expression of SPTAN1 were enriched in UV response down (Fig. 8B). Apical surface, Cholesterol homeostasis and UV response down were also significantly enriched in samples with high expression of CDC16 (Fig. 8C). Coagulation, interferon alpha response and interferon gamma response gene sets was significantly enriched in samples with high expression of RBBP6 (Fig. 8D). It has been shown that dysregulation of lipid metabolism is associated with aging, alterations in lipid rafts and brain lipid peroxidation levels[43]. Our results showed the core role of KDELR1, SPTAN1, CDC16 and RBBP6 in the lipid metabolism and immune response.

## Construction of key gene-related mRNA-miRNA-lncRNA network

**Fig. 9:**
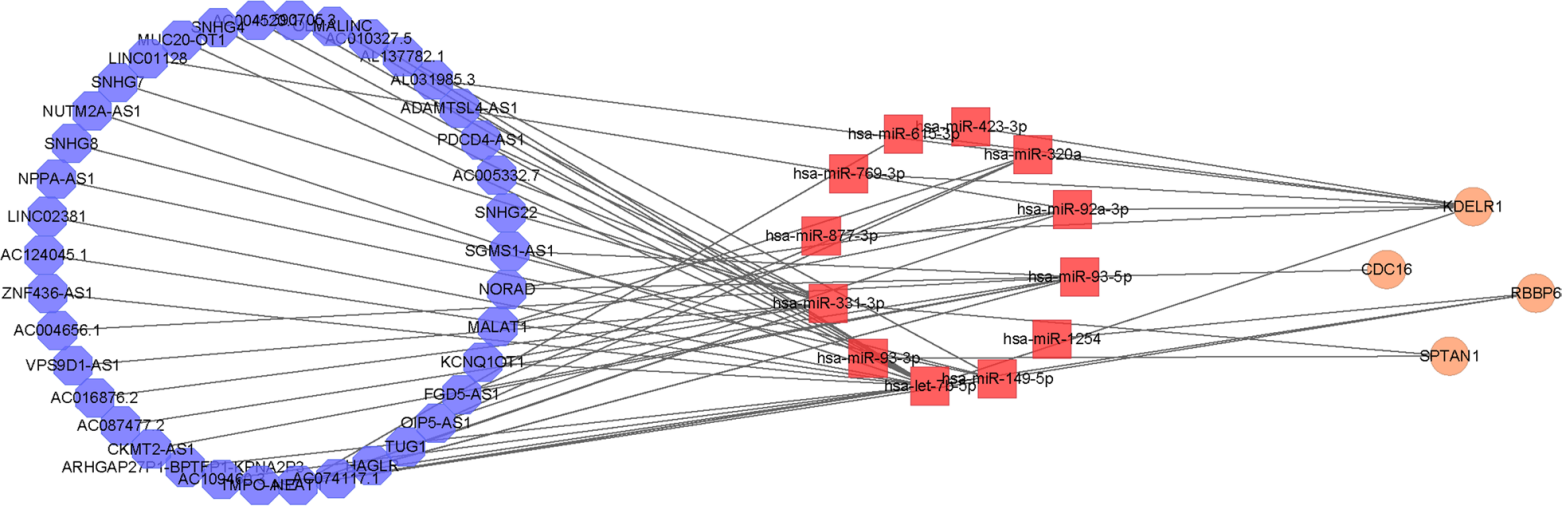
The mRNA-miRNA-lncRNA regulatory network of 4 key genes

For a further understanding of the role of key genes in AD occurrence, we built mRNA-miRNA-lncRNA network based the four key genes. Firstly, 12 miRNAs interacting with key genes were found in the multiMiR database based on four key genes KDELR1, SPTAN1, CDC16 and RBBP6, and then 38 lncRNAs interacting with 12 miRNAs were identified in the starBase database based on our screening criteria (Fig. 9). Thus, we obtained the mRNA-miRNA-lncRNA regulatory network of 4 key genes (containing 54 nodes and 60 edges). These interacting RNAs may be key mechanisms affecting the pathogenesis of AD.

## Discussion

Microglia are the brain-resident immune cells, and many studies regards Aβ-associated mononuclear phagocytes as microglia [44]. There are now evidences that blood-derived monocytes can infiltrate the brain of AD patients through the BBB [45, 46]. In cell cultures incorporating Aβ42, the percentage of monocytes/macrophages (M/M) is significantly higher and M/M express chemokines to promote their migration through the BBB [47]. Monocytes recruited in the brain can phagocytose Aβ in the brain parenchyma [48]. In addition, not only Aβ in central nervous system can be removed, but also Aβ that spreads from the brain to the periphery can be captured and phagocytosed by peripheral monocytes. In this study, we analyzed transcriptomic data from hippocampus of AD patients in GEO database. We revealed the difference immune cell types in hippocampus between AD patients and healthy controls. In addition, we identified the pink and green modules are the key modules closely related to AD.

Based on the PPI network and cytoHubba, we identified 4 key genes associated with monocytes and AD, including KDELR1, SPTAN1, CDC16 and RBBP1, and found that these 4 genes differentially expressed in 5XFAD transgenic mice and WT mice. The GSEA and mRNA-miRNA-lncRNA network based on these 4 key genes further confirmed the possibility of these key genes affecting AD.

KDELR1, KDEL endoplasmic reticulum protein retention receptor 1. It could regulate integrated stress responses (ISR), and promote the naive T-cell survival in vivo [49], and regulates T-cell homeostasis through PP1 (protein phosphatase) [50]. KDELR1 is also one of the candidate molecules associated with neurodevelopmental disorders [51], suggesting it may be one of the key molecules associated with the occurrence of AD. SPTAN1, spectrin alpha, non-erythrocytic 1, is essential for myelin formation [52]. Patients with SPTAN1 mutations have also been found to present with peripheral neuropathy, severe dyslexia, and executive function difficulties [53]. SPTAN1 is downregulated in the hippocampus of patients with medial temporal lobe epilepsy(MTLE), which is usually involved in drug-resistant seizures and cognitive deficits[54]. Therefore, we believe that SPTAN1 is also a key potential molecule associated with Alzheimer’s disease. CDC16, cell division cycle 16, functions as a protein ubiquitin ligase. Together with other proteins, CDC16 forms a protein complex containing the Tre2-Bub2-Cdc16 (TBC) structural domain, the protein that belongs to the Rab-specific GTPase-activating protein (GAP) and is highly conserved in eukaryotes [55]. The TBC and LysM Domain containing (TLDc) proteins containing the structural domain of TBC1 domain family member 24 (TBC1D24) are associated with neurodevelopmental disorders and are mainly involved in the oxidative stress response [56, 57]. Therefore, we speculate that CDC16 may also be one of the key molecules affecting neurodevelopment in AD. RBBP6, retinoblastoma binding protein 6. In various human cancers, RBBP6 is involved in the regulation of cell cycle and apoptosis [58]. However, the role of RBBP6 has not been studied in AD, and it may be a new target related to AD pathology. What’s more, we performed the GSEA and mRNA-miRNA-lncRNA regulatory network to have a more comprehensive knowledge of the roles of key genes in AD.

To sum up, the current study initially assessed the abundance of immune cells in the hippocampus and identified monocytes were associated with AD. We identified and verified 4 key genes associated with the occurrence of AD by multiple methods, and also revealed the signaling pathways associated with immune response in AD, which might provide new insights for immunological studies in AD pathology.

## Supplementary Information

**Additional file 1: Fig. S1.** The selection of the soft-thresholding power β.

## Data Availability

All data generated or analyzed during this study are included in this published article. The datasets generated and/or analyzed during the current study are available in the GEO repository, https://www.ncbi.nlm.nih.gov/geo/, (GSE5281, GSE48350). We have deposited all the code used in this study and the input data for each figure on the Figshare website. All R code for the manuscript: 10.6084/m9.figshare.22014869.v2. All the input data for each figure: 10.6084/m9.figshare.22014806.v1.

## Abbreviations

AD: Alzheimer’s disease
GEO: Gene expression omnibus
RF: Random forest
BBB: Blood brain barrier
Aβ: Amyloidβ
ssGSEA: Single sample gene set enrichment analysis
WGCNA: Weighted gene co-expression network analysis
GS: Gene significance
MM: Module importance
GO: Gene ontology
KEGG: Kyoto encyclopedia of genes and genomes
ISR: Integrated stress responses
PPI: Protein–protein interaction
STRING: Search tool for the retrieval of interacting genes
ROC: Receiver operating characteristic
GSEA: Gene set enrichment analysis
AUC: Area under curve
ISR: Integrated stress responses
PP1: Protein phosphatase 1
MTLE: Medial temporal lobe epilepsy
TBC: Tre2-Bub2-Cdc16
GAP: GTPase-activating protein
TLDc: TBC and LysM Domain containing
