# Supplementary figures and images for: Identification of immune cells infiltrating in hippocampus and key genes associated with Alzheimer’s disease
Source: BMC Med Genomics. 2023 Mar 13;16:53. doi: 10.1186/s12920-023-01458-2 (PMC10009990; doi:10.1186/s12920-023-01458-2)

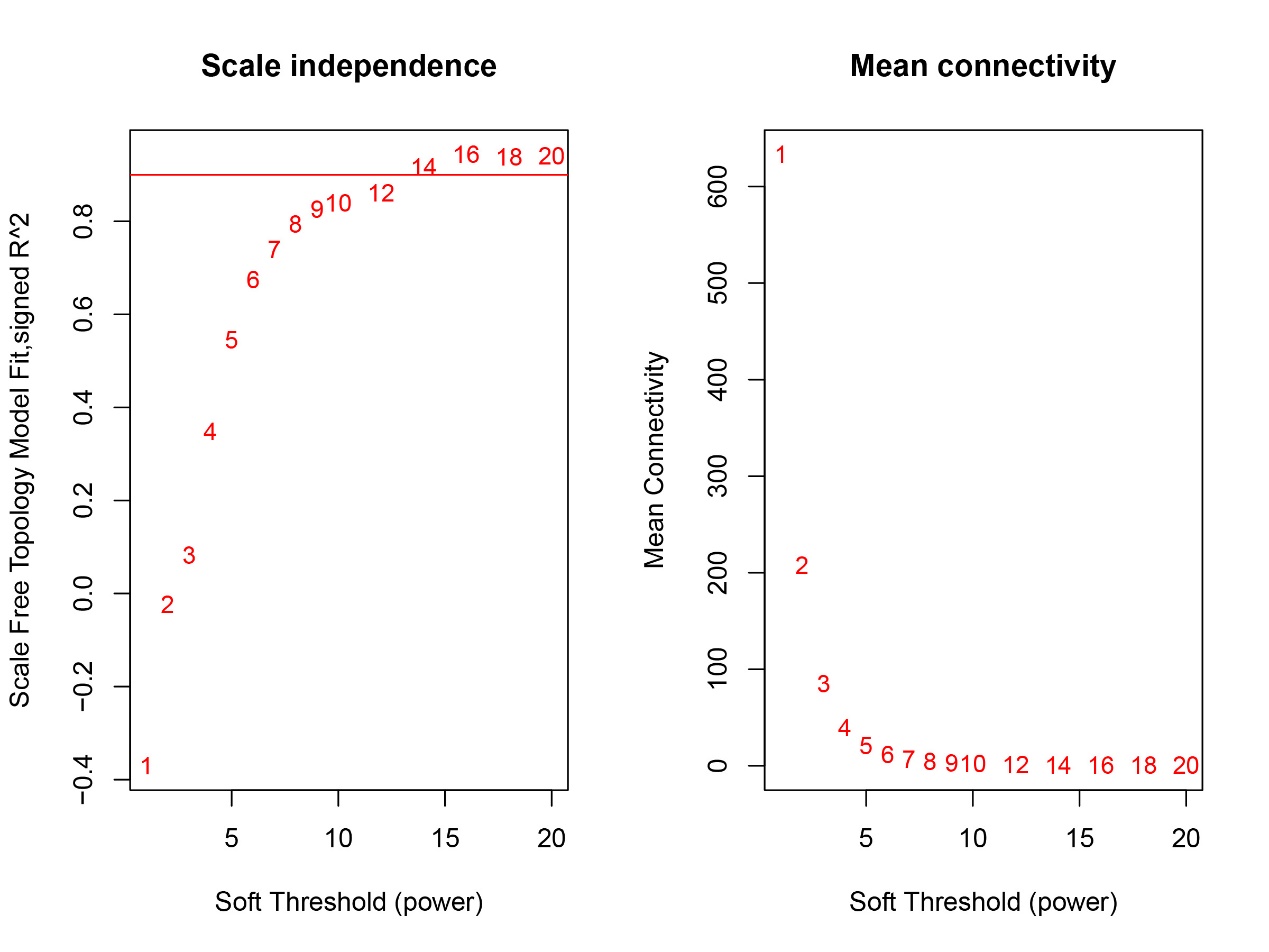


**Supplementary Figure 1.** The selection of the soft-thresholding power β.

Supplement: Supplementary file 1 — Additional file 1: Fig. S1. The selection of the soft-thresholding power β. [file 12920_2023_1458_MOESM1_ESM.docx]
